# EMQN best practice guidelines for genetic testing in dystrophinopathies

**DOI:** 10.1038/s41431-020-0643-7

**Published:** 2020-05-18

**Authors:** Carl Fratter, Raymond Dalgleish, Stephanie K. Allen, Rosário Santos, Stephen Abbs, Sylvie Tuffery-Giraud, Alessandra Ferlini

**Affiliations:** 1 Oxford Medical Genetics Laboratories, Oxford University Hospitals NHS Foundation Trust, Oxford, UK; 2 Genomics Quality Assessment (GenQA), Edinburgh, UK; 3 Department of Genetics and Genome Biology, University of Leicester, Leicester, UK; 4 West Midlands Regional Genetics Laboratory, Birmingham Women’s and Children’s NHS Foundation Trust, Birmingham, UK; 5 Unidade de Genética Molecular, CGMJM, Centro Hospitalar Universitário do Porto, Porto, Portugal; 6 Genetics Laboratories, Cambridge University Hospitals NHS Foundation Trust, Cambridge, UK; 7 Laboratory of Genetics of Rare Diseases (LGMR), University of Montpellier, Montpellier, France; 8 Unit of Medical Genetics, Department of Medical Sciences, University of Ferrara, Ferrara, Italy; 9 Dubowitz Neuromuscular Unit, University College London, London, UK

**Keywords:** Genomics, Genetics, Genetic testing

## Abstract

Dystrophinopathies are X-linked diseases, including Duchenne muscular dystrophy and Becker muscular dystrophy, due to *DMD* gene variants. In recent years, the application of new genetic technologies and the availability of new personalised drugs have influenced diagnostic genetic testing for dystrophinopathies. Therefore, these European best practice guidelines for genetic testing in dystrophinopathies have been produced to update previous guidelines published in 2010.

These guidelines summarise current recommended technologies and methodologies for analysis of the *DMD* gene, including testing for deletions and duplications of one or more exons, small variant detection and RNA analysis. Genetic testing strategies for diagnosis, carrier testing and prenatal diagnosis (including non-invasive prenatal diagnosis) are then outlined. Guidelines for sequence variant annotation and interpretation are provided, followed by recommendations for reporting results of all categories of testing. Finally, atypical findings (such as non-contiguous deletions and dual *DMD* variants), implications for personalised medicine and clinical trials and incidental findings (identification of *DMD* gene variants in patients where a clinical diagnosis of dystrophinopathy has not been considered or suspected) are discussed.

## Introduction

Dystrophinopathies are X-linked genetic diseases due to dystrophin (*DMD*, OMIM *300377, HGNC ID: 2928) gene variants. The main phenotypes associated with pathogenic *DMD* variants are severe Duchenne muscular dystrophy (DMD, OMIM #310200), milder Becker muscular dystrophy (BMD, OMIM #300376) and isolated cardiac involvement leading to the X-linked dilated cardiomyopathy (XLDC, OMIM #302045). Rare phenotypes, such as quadriceps myopathy or isolated high serum creatine kinase (CK) levels (‘hyperCKaemia’), have also been described [1–3].

In all dystrophinopathy phenotypes, males have a hemizygous (or rarely mosaic) pathogenic *DMD* variant. Heterozygous females can be asymptomatic carriers, although in some cases they present symptoms ranging from adult-onset mild muscle weakness and/or dilated cardiomyopathy to rare instances of a DMD- or BMD-like phenotype [4]. The rate of occurrence of new pathogenic variants in the *DMD* gene is high, and a significant proportion of cases arise de novo; there is also a relatively high frequency of germline mosaicism [5].

*DMD* is the largest human gene in terms of genomic length, spanning 2.2 Mb. It is an ancient gene with some regions very well conserved across species [6], and the oldest region at the 3′ end with homology to the Sea urchin *DMD* short gene. In humans, it comprises 79 constitutive exons encoding the muscle isoform (Dp427m), plus 6 other alternative first exons corresponding to the two full-length isoforms mainly expressed in brain cortex (Dp427c) and cardiac Purkinje cells (Dp427p) and to the four shorter 3′ isoforms that are expressed in the retina (Dp260), brain (Dp140), peripheral nerve (Dp116) and general or ubiquitous (Dp71). All variant types may occur in the *DMD* gene: large deletions (68%), large duplications (10%), small variants (i.e. missense, nonsense, insertion, deletion, indel and splicing variants) (22%) and atypical variants including deep intronic variants and complex intragenic rearrangements (<1%) (Table 1).

**Table 1:** Spectrum of *DMD* pathogenic variants.

Phenotype	Dystrophinopathy (all)	DMD	BMD
Deletion (≥1 exon)	68%	61–66%	80–81%
Duplication (≥1 exon)	10%	11–13%	6–9%
Complex rearrangements	<0.5%	<0.5%	<0.5%
Total CNV	78%	74–77%	87–89%
Nonsense	9%	12–13%	3%
Frameshift	7%	6–8%	2%
Missense	0.40%	0.3–0.9%	0.5–0.7%
Splicing (≤10 bp from intron)	5%	4–5%	5–7%
Splicing (mid/deep intronic)^a^	0.6%^a^		
Total small variants	22%	23–26%	11–13%

Distribution of *DMD* pathogenic variants in European families with dystrophinopathy [49, 95]. These figures are approximate and may differ slightly in other populations.

^a^Deep and mid intronic splicing variants have been estimated to account for 0.2–1% of pathogenic variants in the *DMD* gene [61]; this is the only class of pathogenic variant that is not expected to be detected by combined Copy Number Variation (CNV) analysis and routine sequencing of the *DMD* gene.

Genetic testing for dystrophinopathies is recommended as part of routine clinical practice since a genetic diagnosis allows: confirmation of a clinical diagnosis ensuring appropriate care and follow up; access to personalised treatments; carrier identification and family planning. Where possible, genetic testing should be undertaken in laboratories accredited for clinical diagnostic testing to an appropriate standard, such as ISO15189.

Previous guidelines on molecular diagnostics in DMD/BMD were published in 2010 [7]. Both the application of new technologies (particularly next generation sequencing) in diagnostics and the availability of new personalised drugs have led to consensus that new and updated guidelines are required. The guidelines presented here constitute a revision of the previous guidelines, coordinated by authors of the 2010 guidelines and the European Molecular Genetics Quality Network (EMQN).

The recent approval and establishment of European Reference Networks (ERNs) across European countries has primed large collaborative initiatives devoted to Rare Disease (RD) care and cure across European Union member states. ERN establishment also stresses the concept of excellence-in-health for RD care, ensuring that trained and skilled centres maximise diagnostic success, appropriate care and follow up for RDs. Among ERNs, the EURO-NMD ERN [8] is dedicated to neuromuscular diseases, including dystrophinopathies, and its mission is also to prime revision of guidelines and to promote care equality across countries, greatly reducing the patient burden due to delay or absence of genetic diagnosis.

Based on previously published experiences and given the deep psychological and socio-economic implications, the issue of newborn screening is not included in these guidelines. We believe that a dedicated forum would be required for this topic to be fully discussed and developed.

## Methods

The original guidelines were based on a meeting of 29 senior scientists from Europe, the USA, India and Australia, held in Naarden, The Netherlands in November 2008 to establish consensus best practice guidelines for molecular diagnosis of Duchenne and Becker muscular dystrophy [7].

An update of the guidelines was deemed necessary due to the rapidly evolving scientific nature of genetic testing since 2008. To achieve a broad expert consensus, a drafting group of European-based experts, including authors from the original guidelines, compiled evidence to support updated recommendations for genetic testing and reporting of dystrophinopathies culminating in the first draft version of the guidelines in March 2019. The guidelines were then disseminated to a global network of molecular geneticists and clinicians from a list of 135 laboratories who currently participate or have historically participated in the EMQN-organised scheme for DMD between 3rd April 2019 and 1st May 2019 for consultation and amendments. The guidelines were also distributed to known UK diagnostic labs involved in *DMD* testing for comments. The feedback was collected and appraised by the expert drafting group and the draft document was amended accordingly. The draft document was then reviewed by The EMQN Management Group who made suggestions for clarity and content improvements that were appraised by the expert drafting team. In October 2019 the document was amended in order to formulate the final version of the guidelines.

## Results

### Variant detection—technical aspects

#### Deletions and duplications detection (Level 1 testing)

**Fig. 1:**
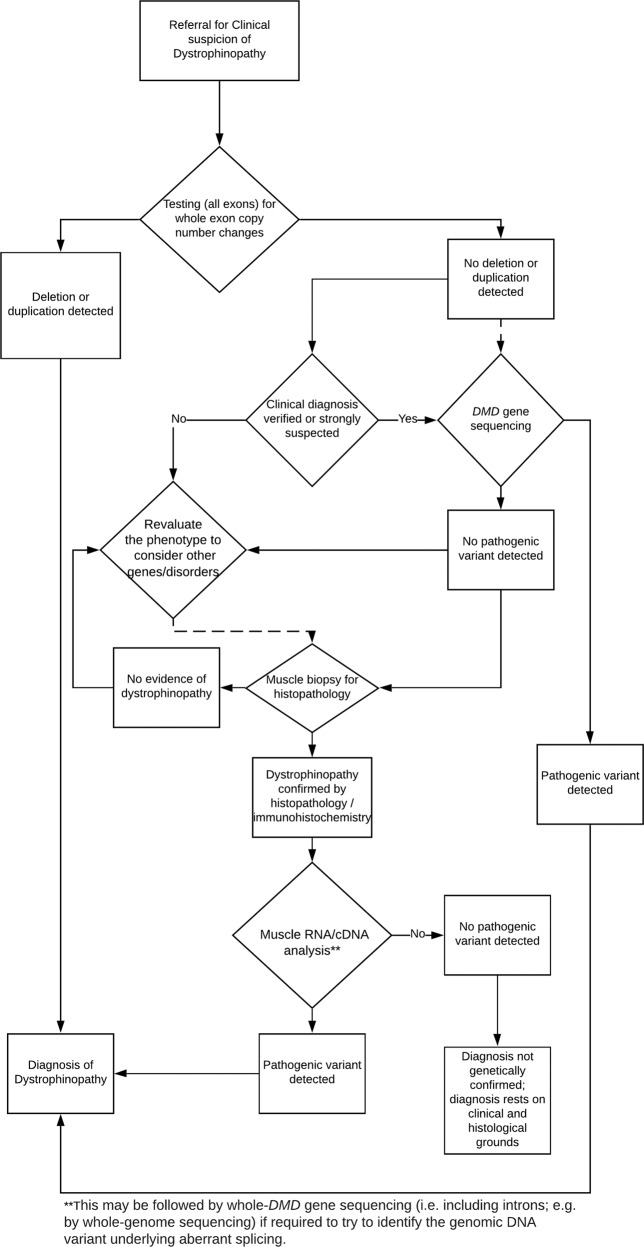
Flowchart of the recommended molecular diagnostic algorithm for dystrophinopathy.

Since whole-exon deletions or duplications are the predominant type of pathogenic variant in the *DMD* gene (~78%; Table 1), an initial screen which detects the majority of these copy number variations (CNVs) should be the first diagnostic test offered (refer to Genetic testing strategy section and Fig. 1). The detection of exon CNVs is based on quantitative methods that allow determination of the relative copy number of all exons within the *DMD* gene.

Among the many quantitative methods available, multiplex ligation-dependent probe amplification (MLPA), is currently the most widely applied as an initial diagnostic test in laboratories due to the convenience, high sensitivity [9], reduced cost and commercial availability of an assay that is CE-marked for in vitro diagnostic use (MRC Holland SALSA MLPA Probemixes P034 and P035). MLPA only determines the extent of deletions or duplications to the exon level of resolution. The kits are designed to contain only a single probe for each of the 79 exons of Dp427m plus exon 1 of the Dp427c isoform. Hence the reliability of MLPA results is high for CNVs involving multiple adjacent probes/exons, but less so if an apparent single-exon deletion or duplication is identified. Therefore, the result must be confirmed using an independent method (quantitative, such as real-time PCR, or qualitative, such as Sanger sequencing, for example) if MLPA identifies an apparent single-exon deletion or duplication. Multiplex PCR or microsatellite marker analysis are possible methods to confirm single-exon deletions in males (by non-amplification of a PCR product or microsatellite marker alongside appropriate internal amplification controls). An independent method will determine whether the initial result could have been caused by a sequence variant (e.g. pathogenic or non-pathogenic) inducing an allele dropout by preventing hybridisation of a MLPA probe [10], or for duplications, if the result was an anomaly. Repeating the MLPA assay may also be appropriate for single-exon duplications. Although rare, false-negative signals will be obtained if the probe does not map in the mutated region (i.e. partial exonic deletion) [11].

Among other quantitative full-gene approaches, the oligonucleotide-based array comparative genomic hybridization (CGH) technique has proved to be very efficient in detecting CNVs in the *DMD* gene [12, 13]. High-resolution array CGH uses thousands of oligonucleotide probes to interrogate copy number across the entire 2.2 Mb genomic region of the *DMD* gene including all exons and introns as well as 5′ and 3′ flanking regions, and thereby maps rearrangement intronic breakpoints to relatively narrow intervals depending on the spacing of the probes at the breakpoints. Gene-targeted high-density array CGH has a further advantage over most other methods which generate only a single result per exon, since most deletions or duplications are likely to be detected simultaneously by several oligonucleotide probes on the array. This eliminates the possibility of a false positive result due to the presence of a single nucleotide polymorphism (SNP) in a single probe or primer binding site. It also can detect loss or gain of sequences at intronic breakpoints associated with some inversions and complex rearrangements, thereby offering a slightly higher detection rate than MLPA [14, 15].

For both MLPA and array CGH, the information on CNVs gives no insight on the orientation or location of insertions, duplications or rearrangements. Also, these techniques are unable to detect balanced rearrangements. Both techniques can however identify non-contiguous exon deletions or duplications which should be further tested (see Atypical findings below).

Quantitative assays of all *DMD* exons have gradually superseded techniques that interrogate only selected exons as they offer a higher detection rate for CNVs in the *DMD* gene. In particular, they allow the detection of non-contiguous exon deletions, duplications or triplications that occur in this huge gene, whose identification is crucial to provide an accurate diagnosis and genetic counselling [15, 16]. In addition, these techniques can be used for female carrier testing. In this context, the conventional Multiplex PCR assay initially developed to detect the majority of *DMD* deletions in male patients by screening a subset of 18 exons [17, 18] is now hardly ever used as it does not characterise the end points of all deletions, does not detect other CNVs, and cannot be applied to carrier testing. Therefore, it is recommended that conventional Multiplex PCR no longer be used for diagnostic testing and should be replaced by a quantitative assay for all *DMD* exons.

Next generation sequencing (NGS) approaches are now routinely adopted to accurately detect single nucleotide variants (SNVs) (see below) and have emerged as a technology with the capability to detect accurately both SNVs and CNVs in a single assay [19, 20]. However, CNV analysis via NGS is not yet routinely adopted in diagnostics. CNV calls from NGS data depend on high depth and uniformity of coverage across all target sites, and currently available bioinformatics tools are still not sensitive enough to reliably pick up all types of CNVs without expert bioinformatics knowledge and extensive validation [21, 22]. Hence, accurate and robust CNV calling is particularly difficult for female carrier testing and for duplications in males. Therefore, further technical and bioinformatics improvements are still needed, but NGS-based CNV detection will increasingly be adopted into routine diagnostics over the next few years. Noteworthy, both short read and long read whole-genome sequencing are beginning to be implemented by diagnostic laboratories and may prove to be the preferred methods for sensitive and specific detection of both CNVs and SNVs.

When interpreting the deletion or duplication result (see below), if the predicted severity is discrepant with the observed clinical phenotype, it may be useful to repeat the tests on a second sample and/or to carry out additional laboratory tests using different methods, or offering more detail, in order to look for an explanation for the discrepancy. This additional work is not essential for genetic diagnostic purposes and may not be possible in all centres. However, an accurate determination of the extent of a deletion or a duplication and of its potential impact on transcripts may be required for the inclusion of a patient in a genotype-based therapy.

#### Small variants detection (Level 2 testing)

The term ‘small variants’ is used here to collectively describe missense, nonsense, small insertion and deletion, indel and splicing variants. As pathogenic small variants in the *DMD* gene are mostly private and are distributed all along the gene, all 79 exons and flanking intronic sequences should be analysed. This is usually performed by DNA sequencing, but sequencing of RT-PCR derived cDNA can also be carried out. Until recently, the Sanger method (first-generation sequencing) was the most popular and routine technique used to individually sequence all *DMD* exons [23]. Sanger sequencing is highly accurate but of course highly time consuming, therefore the new NGS approaches have led to a rapid change in sequencing strategies. In comparison with Sanger sequencing, which can only be performed on one target of a few hundred nucleotides (typically one exon) per reaction, NGS enables a large number of targets to be sequenced in parallel with deep sequence coverage in multiple patients at one time. While whole-*DMD* gene sequencing including all exons, introns and promoter has been reported [24], current approaches are mostly amplicon-based targeted NGS to sequence all 79 exons and intronic flanking sequences of the *DMD* gene [19, 20]. Whole-exome sequencing and whole-genome sequencing, with targeted analysis of the *DMD* gene, are also increasingly being adopted [25, 26]. Data analyses are performed using in-house pipelines or commercially available software. Thus, NGS offers numerous technical advantages (improved cost-effectiveness, scalability, resolution) and can enhance the detection of low-level somatic mosaicism in patients or probands’ mothers as compared to Sanger sequencing. High quality sequence read depth must be sufficient across the region analysed, and laboratories may need to carry out Sanger sequencing of any regions falling below their validated minimum read depth.

However, while NGS has become more widely used to screen for unknown variants, Sanger sequencing remains the standard method used for known familial variant testing, validation of variants identified by NGS and prenatal diagnosis.

#### RNA analysis (Level 3 testing)

In patients with an ascertained clinical diagnosis of dystrophinopathy but no CNVs or small variants identified, RNA-based methods offer a valuable tool with a high likelihood of being able to detect variants that escape detection using level 1 and 2 DNA approaches, such as complex rearrangements or deep intronic variants leading to pseudo-exon insertion or cryptic splice site recognition in the mature transcripts. These variants appear to be of low frequency (Table 1) [27]. Conventional Sanger sequencing can be performed on RT-PCR derived cDNA from muscle RNA. Alternatively, a microfluidic card tool based on a TaqMan assay can be used to profile the entire *DMD* mRNA including all exon–exon junctions, and it is able to identify multiple splicing anomalies [28]. Although pathogenic variants have been identified in *DMD* mRNA from blood [29, 30], only low level illegitimate *DMD* transcription occurs in lymphocytes, so analysis is technically challenging and any aberrant splicing may not reflect splicing in muscle. Recently, alternative sources of *DMD* transcripts have emerged such as stem cells [31] or extracellular RNA [32] from urine that could help overcome the difficulty of obtaining muscle biopsies in some patients.

If a pathogenic variant is identified in cDNA, it should be followed by sequencing of the appropriate region(s) in genomic DNA to confirm the result and allow future genomic-based testing in relatives. Similarly, when an intronic region corresponding to a pseudo-exon or the use of a new intronic splice site is found in RT-PCR derived cDNA, the genomic region surrounding the inserted sequence should be sequenced to identify the causative intronic variant. In some rare occasions, it may not be straightforward or even possible to characterise the sequence change at the genomic level responsible for the detected structural change in the transcript.

In the near future, targeted RNA-seq approaches, whole-transcriptome sequencing and/or whole-genome sequencing are likely to be used to explore unsolved dystrophinopathy cases to identify variants causing aberrant transcripts.

RNA analysis is also useful to accurately define the splicing outcomes of DNA variants, to establish whether genomic duplications are in direct tandem orientation, together with their consequences on the mRNA, or to further explore genotype-phenotype discrepancies. Altered splicing events (e.g. exon skipping, partial intron retention, pseudo-exon insertions) may occur that change the predicted impact on the reading frame of genomic rearrangements or exonic variants (e.g. exon skipping-associated nonsense variants).

### Genetic testing strategy

#### Diagnostic testing in male patients

Affected males suspected of having a dystrophinopathy are referred for genetic confirmation of the clinical diagnosis. Typically, dystrophinopathy is suspected on the basis of clinical symptoms and high serum creatine kinase (CK) levels. In some cases, family history may also be suggestive of a dystrophinopathy. Molecular testing is usually now requested prior to a muscle biopsy, given the high frequency of deletions/duplications of one or more exons and new high-throughput NGS methods for *DMD* gene sequencing, but in some centres or in some cases a muscle biopsy may still be needed before any genetic testing is carried out.

Genetic confirmation of a dystrophinopathy is achieved by demonstrating the presence of a clearly pathogenic variant in the *DMD* gene. A flowchart of the recommended molecular diagnostic algorithm is shown in Fig. 1. Since whole-exon deletions or duplications account for ~78% of pathogenic variants (Table 1), testing all exons of the *DMD* gene for whole-exon deletions and duplications is the first genetic diagnostic test recommended. If this analysis does not identify a pathogenic deletion or duplication and if dystrophinopathy is strongly suspected, then sequencing of the entire coding region of the *DMD* gene is recommended. It is not expected that *DMD* sequencing is available in every laboratory, but laboratories in which it is not available should seek an arrangement with another laboratory to carry out this analysis. If a pathogenic variant is still not identified, then a muscle biopsy and dystrophin analysis by immunohistochemistry (and western blot where possible) will be needed to establish a precise clinical diagnosis. In these cases, further molecular genetic analysis by muscle cDNA sequencing can be undertaken by specialist centres and will usually identify a pathogenic variant. Therefore, >99% of genetic diagnosis of dystrophinopathy is achievable using the entire cohort of testing (1st, 2nd and 3rd level). Nevertheless, in those rare cases with positive muscle biopsy proving a quantitative or qualitative dystrophin abnormality but without pathogenic genetic variant identified, a clinical diagnosis of dystrophinopathy should be concluded, since no molecular testing protocol can currently demonstrate 100% sensitivity. This should be taken into account as very important for Rare Disease certification.

If a pathogenic *DMD* variant is not identified by analysis for whole-exon deletions and duplications or after *DMD* gene sequencing, then in some cases alternative diagnoses should be considered, depending on the available clinical evidence and test results. Possible alternatives, especially for patients with milder clinical severities (for example, those referred with suspected BMD) but also for patients referred with DMD at an early age, might include a limb girdle muscular dystrophy, or Emery–Dreifuss muscular dystrophy.

For patients with additional symptoms not normally explained by a dystrophinopathy, such as dysmorphic features or severe learning difficulties, the possibility of a contiguous gene syndrome should be considered when a deletion/duplication extending to at least the first or last exon of the *DMD* gene has been identified. Therefore, array CGH testing is recommended as an additional first line test for these patients.

For some referrals for diagnostic confirmation in patients with a family history of dystrophinopathy, the familial pathogenic variant may already be known. Specific testing for the familial pathogenic variant is sufficient for these patients. However, if the familial variant is not detected, then further testing should be considered following the diagnostic algorithm in Fig. 1 as, although rare, the occurrence of independent new pathogenic variant events in related patients through either the paternal or maternal lines has been reported [33].

From the patients' and relatives' perspectives, the speed with which a diagnosis can be made is extremely important to minimise anxiety and to reduce the risk of recurrence of the disease in the family, as well as for variant-specific (personalised medicine) approved treatments and enrolment in clinical trials. Therefore, the above workflow should always be carried out in a timely manner.

#### Diagnostic testing in manifesting female patients

Although the classic dystrophinopathy phenotypes of DMD and BMD typically affect males, females can present with DMD-, BMD-like or milder phenotypes, such as dilated cardiomyopathy and/or mild muscle weakness [4]. As dystrophinopathy is less well recognised in females and symptoms may be similar to other limb girdle muscular dystrophies, manifesting females may not receive an accurate genetic diagnosis.

Most manifesting females are heterozygous for a pathogenic *DMD* variant and have a milder phenotype than DMD males. Clinical manifestation in these cases is most likely due to skewed X-inactivation. The recommended testing strategy in these manifesting females is equivalent to that outlined above (and in Fig. 1) for males, i.e. CNV analysis and sequencing if a diagnosis is strongly suspected, followed by muscle biopsy (enabling RNA analysis as appropriate) if no diagnosis is made.

A small proportion of manifesting females have a DMD phenotype and are sometimes referred to as ‘DMD-like’ girls. Additional testing is appropriate in these cases. Karyotyping is recommended due to the possibility of chromosomal aberrations, particularly balanced X;autosome translocations, leading to 100% skewed X-inactivation of the normal X-chromosome and hence the DMD phenotype [34]. Girls with Turner syndrome (45,X) and DMD have also been reported [35]. There are also other extremely rare cases of DMD-like girls: cases without chromosomal translocation but presenting with complete skewed inactivation of the normal X-chromosome in skeletal muscle (38, STG personal communication); girls with two pathogenic variants (4, AF personal communication); girls exhibiting uniparental disomy with a pathogenic *DMD* variant; or coexistence of a pathogenic *DMD* variant with a pathogenic androgen receptor variant in a phenotypically female individual with a 46,XY karyotype [35]. Therefore, in the absence of a chromosomal aberration, these possibilities should be considered.

As with diagnostic testing in males, in some manifesting females with a family history of dystrophinopathy, the familial pathogenic variant may already be known, in which case specific testing for the familial pathogenic variant is usually sufficient.

### Carrier testing in females

Most females referred for molecular testing are clinically asymptomatic individuals with a family history of dystrophinopathy in males. Testing is undertaken to determine whether the individual is a carrier of dystrophinopathy, and hence this analysis is referred to as carrier testing. However, it is important to be aware that a significant proportion of ‘carrier’ females develop some dystrophinopathy-related symptoms during their lifetime (refer to ‘Reporting results’ section).

Carrier testing is important to allow adequate and appropriate family planning, which may lead to prenatal testing, including non-invasive prenatal diagnosis (NIPD), or preimplantation genetic diagnosis. Individuals undergoing carrier testing should always receive appropriate genetic counselling prior to testing.

#### Carrier testing for known familial pathogenic variant

When the familial pathogenic variant is known, carrier testing should be undertaken by specific testing for this variant. Whenever possible, a sample from the index case (or a known carrier) should be run as a control sample or (at least) a written laboratory report describing the variant of the index patient should be available to avoid data transmission problems. If the familial pathogenic variant is a single-exon deletion or duplication, then preferably the testing method should not rely on a single probe or primer pair; therefore, where possible, two independent/complementary tests (e.g. MLPA and Sanger sequencing, or MLPA and microsatellite analysis, for a single-exon deletion), or a single test that interrogates multiple loci within the deletion or duplication (e.g. high density array CGH), should be performed if there is a possibility of a false result due to non-amplification or non-hybridisation.

If the variant in the index case cannot be found in genomic DNA from his mother, the frequency of germline mosaics still confers a significant recurrence risk for future children (see ‘Reporting results’ and ‘Atypical findings’ sections below).

In exceptional cases when the *DMD* gene defect is detectable only at the RNA level, the need to perform RNA analysis from a muscle biopsy in female relatives can be considered (or haplotype analyses performed if applicable, see ‘Carrier testing by haplotype analysis’ section).

#### Carrier testing when the familial pathogenic variant is unknown (no available proband)

When the familial pathogenic variant is unknown and an affected male is not available to be tested, female relatives at risk of being carriers should be offered the full cohort of level 1 and 2 genetic testing (i.e. CNV analysis and sequencing) since these two approaches are cost effective and offer ~99% sensitivity.

Testing should start with the woman who has the highest prior carrier risk. Ideally this would be an obligate carrier, but more commonly this is the mother of an index case, and in many cases only a more distant relative (with a lower carrier risk) is available. Measurement of serum CK levels, ideally in all at-risk females belonging to the pedigree, may also be helpful in refining carrier risk prior to or alongside genetic testing. Appropriate genetic counselling prior to genetic testing is particularly important for these referrals (in view of the residual risks if a pathogenic variant is not detected).

If no pathogenic variant is detected by level 1 and 2 genetic testing, a muscle biopsy in the at-risk female (especially for obligate carriers or females with repeatedly high CK) might be appropriate to evaluate dystrophin expression and for RNA studies. If not possible/available, urine stem cell RNA studies might be helpful to identify atypical *DMD* variants.

#### Carrier testing by haplotype analysis

Haplotype analysis, also known as linked marker analysis, is an alternative molecular testing approach which may be appropriate in very rare cases, if key family members are available and the family structure is suitable. The rare instances when this analysis may be useful include when there is very strong evidence of dystrophinopathy (e.g. classic clinical signs, elevated serum CK levels and ideally supportive muscle immunohistochemistry) and no pathogenic variant is detected in the proband (including when a variant of uncertain clinical significance is identified in one or more members of the family) or when it has not been possible to identify at the genomic level the cause of a structural change detected at the RNA level in the proband (refer to RNA analysis (Level 3 testing)). Carrier risk is determined by tracing the inheritance of high and low risk *DMD* haplotypes within a family. Polymorphic microsatellite markers and/or SNPs are used for the analysis. A large resource of intragenic and flanking markers is maintained on the Leiden Muscular Dystrophy pages website [36]. Multiple informative markers should be used, which span the length of the gene and closely flank the gene at each end. This is important due to the high probability of recombination across the *DMD* gene. Individual haplotypes are then constructed from these data. The results should be interpreted and reported with care, particularly if the diagnosis is in any doubt (see ‘Reporting results’ section for further information).

### Prenatal diagnosis, NIPD and PGT

Considering the World Health Organization (WHO) document on Quality & Safety in Genetic Testing [37] under the principle of clinical utility, prenatal diagnosis for DMD/BMD/XLDC or any dystrophinopathy phenotype that is clinically relevant should be limited to male pregnancies only. Indeed, at present it is not possible to predict whether a female heterozygous for a *DMD* pathogenic variant will manifest any signs of the disorder or not. Hence it is not generally considered appropriate to offer prenatal diagnosis for a female foetus. Nevertheless, prenatal testing of a female foetus may be considered in some rare circumstances, such as documented familial recurrence of X;autosome translocation, documented familial complete skewed X-inactivation, or emotional and/or social motivations that are not directly linked to the *DMD* genotype (e.g. the presence of a DMD-like girl in the family). The familial pathogenic variant will preferably be known in advance of testing a pregnancy and should be confirmed before the prenatal test.

#### Prenatal diagnosis

Invasive prenatal genetic testing for dystrophinopathies is preferentially performed on tissue (placental biopsy) from chorionic villus sampling (CVS) in early gestation (11–12 weeks). High DNA quality and yield is typically obtained from CVS, allowing a wide variety of possible testing methods (including NGS); therefore, results can be obtained and reported rapidly (normally by 2–12 days from sampling). Amniocentesis, typically undertaken at around 15–17 weeks gestation, may be used in rare cases, but has the disadvantages both of being taken later in the pregnancy and of lower DNA quality and yield from direct amniotic fluid; therefore, amniocyte miniculture may be required (at least 5 more days) to obtain sufficient high-quality DNA.

Testing of CVS or amniocentesis is carried out by standard procedures. The known familial *DMD* gene pathogenic variant can be tested using the appropriate technique (CNV detection or sequencing, others for rare atypical variants) that was previously used to identify/verify the variant in the proband. A check for maternal cell contamination (MCC) must be carried out, since its presence at a significant level may affect the interpretation of the foetal result. This can be achieved by the analysis of intragenic *DMD* microsatellite markers that will also allow confirmation of foetal sex and the result of direct mutation testing (haplotype analysis). Alternative methods are also acceptable. It is important to confirm foetal sex by testing the CVS or amniocentesis material regardless of whether non-invasive foetal sexing was previously carried out. Further details and recommendations for checking for MCC in prenatal samples can be found in the ACGS Best Practice Guidelines [38].

#### Non-invasive foetal sexing

It is now possible to non-invasively determine foetal sex during pregnancy, and this testing should be offered to patients as an alternative to chorionic villus sampling or amniocentesis as it will negate the risk of an invasive test where the foetus is female. Non-invasive foetal sexing is possible from 7 to 9 weeks gestation depending on local laboratory policies, and is based on the detection of Y-chromosome specific sequences in the maternal blood during pregnancy [39, 40]. It is important that an ultrasound scan be performed to accurately date and confirm a singleton pregnancy. Testing has limited benefit in twin pregnancies, but may be helpful in the scenario where both foetuses are demonstrated to be female as shown by complete absence of Y-chromosome specific sequence. It is important however that the test is validated on twin pregnancies. Testing is usually carried out using real-time PCR for Y chromosome markers such as *SRY*, with a housekeeping gene such as *CCR5* as a control. There is a test failure rate of up to 5% due to low foetal fraction, and a very small risk of misdiagnosis due to the presence of a vanishing twin (false positive result) or a very low foetal fraction (false negative result).

#### Non-invasive prenatal diagnosis (NIPD)

NIPD for dystrophinopathies is likely to increasingly become feasible using methods such as relative haplotype dosage analysis (RHDO) [41]. RHDO analysis examines SNPs in the cell-free DNA from a maternal blood sample, and shows whether the male foetus has inherited the high risk or low risk haplotype across the *DMD* gene, in comparison to a proband for phasing of the haplotypes. It is likely that new genomic approaches also able to directly detect CNVs will be available shortly due to technical and bioinformatics advances.

#### Prenatal diagnosis by haplotype analysis

In a few cases there may be a diagnosis of dystrophinopathy within the family, but the pathogenic variant has not been identified. Although both CNV detection and sequencing can be quickly performed on the pregnant mother to identify the pathogenic variant in the vast majority of cases, in some rare cases, haplotype analysis using informative polymorphic markers is an option for prenatal diagnosis; this is feasible only if the family structure is suitable and polymorphic markers are informative. Further information on polymorphic markers is included in the carrier testing section above. As with carrier testing, multiple informative markers should be used, which span the entire length of the gene. A decision should be made whether it is appropriate to offer prenatal diagnosis by haplotype analysis prior to prenatal sampling. The results should be interpreted with caution, particularly if the clinical diagnosis is in any doubt (see ‘Carrier testing’ section above and ‘Reporting results’ section for further information).

#### Preimplantation genetic testing (PGT)

PGT is a specialist test carried out in a limited number of centres. For PGT, the same analytical considerations apply as for prenatal testing, but the special requirements of a PGT setting need to be considered. This is detailed in the Best Practice Guidelines from the European Society of Human Reproduction and Embryology Consortium [42]. PGT is therefore beyond the scope of these guidelines. However, it is important to note that this type of genetic diagnosis is appropriate and feasible for dystrophinopathies and is applicable to both CNVs and small variants [43].

### Variant annotation and interpretation

#### Variant annotation

Dystrophinopathies result from sequence variants in the *DMD* gene (HGNC ID:2928). Although the *DMD* gene encodes several transcripts/isoforms, variants are reported in the context of NCBI RefSeq transcript NM_004006.2 which is the transcript (Dp427m) of *DMD* that is predominantly expressed in skeletal and cardiac muscle. The corresponding NCBI RefSeqGene genomic DNA record is NG_012232.1. The Locus Reference Genomic (LRG) [44, 45] reference sequence, LRG_199, is based on these two reference sequences with the transcript being designated t1. No other sequence records are currently supported for the reporting of *DMD* gene sequence variants.

Fully characterised sequence variants should be described in accordance with the Human Genome Variation Society (HGVS) recommendations for description of sequence variants [46]. This includes small sequence variants (e.g. single-nucleotide substitutions and deletions, insertions or duplications of a few nucleotides) and also whole-exon deletions/duplications where the breakpoints have been fully characterised at the genomic DNA level. However, when the breakpoints of whole-exon deletions/duplications have not been defined (e.g. when reporting results of MLPA analyses), use of HGVS variant nomenclature is not required as it is complex and not readily understandable to the referring clinician. It is important that patient reports of whole-exon deletions/duplications include a description that is both meaningful and understandable to the medical community. Therefore, although HGVS variant description recommendations do not refer to the numbering of gene exons, as a variant should never formally be described in the context of exon or intron numbers, it is recommended that all whole-exon deletions/duplications be described with reference to specific exons (e.g. deletion of exons 2–6) in patient reports. Although exon numbering is not controversial for *DMD*, it should be clear from the report how the exons have been numbered; this is most easily achieved by quoting LRG_199t1 as LRGs provide exon numbering.

#### Interpretation of deletions and duplications

Whole-exon deletions or duplications detected in patients with suspected dystrophinopathy can be considered pathogenic in the vast majority of cases.

The correlation of deletions with clinical severity usually depends on their effect on the open reading frame (a reading frame checker is available at the Leiden Muscular Dystrophy pages [36]). Deletions that disrupt the translational reading frame generally lead to complete or near-complete absence of dystrophin protein in muscle, thereby resulting in a severe DMD phenotype in males. In contrast, those that preserve an open reading frame, enabling production of either a reduced amount of normal dystrophin or an altered but partially functional protein (with an intact C-terminus which allows synthesis of the dystroglycan-binding region), generally give rise to a milder BMD clinical presentation [47]. Application of this reading-frame rule to the coding regions of the *DMD* gene thus makes it possible to predict whether a male is likely to develop BMD or DMD [48]. While this holds true for the majority of cases, there are several exceptions, most of which can be explained by splicing choices (e.g. favourable exon skipping of deletion-adjacent exons reframing the translation), lack of some exons encoding vital protein domains (such as those encoding the N-terminus, cysteine rich domain or C-terminus) or alternative translation initiation [11, 49–52]. Indeed, even small in-frame deletions may have a severe pathophysiological consequence; for example, those affecting the amino-terminal actin-binding domain 1 (ABD1), encoded by exons 2–8, or the beta-dystroglycan binding region, encoded by exons 63–70, often result in a severe DMD phenotype [51, 53].

The detection of whole-exon duplications is accepted as being sufficient to confirm a diagnosis in patients with suspected dystrophinopathy. However, it is important to note that diagnostic techniques, such as MLPA or CGH, do not provide information about the location (tandem versus interspersed) or orientation (head-to-tail versus inverted) of the duplicated gene segment. Although most duplications of one or more exons turn out to be direct tandem duplications, in some of these presumed ‘simple’ duplications, studies at the RNA level have revealed unexpected changes such as partial triplications, indels and other complex rearrangements [54, 55]. Additionally, some cases are confounded by splicing choices inducing exon skipping or (partial) intronic sequence retention. It is therefore not surprising that whole-exon duplications have been reported to account for almost one third of the cases behaving as exceptions to the reading frame rule [1]. The possibility of duplications involving extragenic regions (on the X chromosome or on autosomes) due to unequal crossing over and non-homologous end joining is also an issue, as recently reported [56]. Most extreme of all, it is possible, although highly unlikely in an individual with suspected dystrophinopathy, that an apparent duplication could be inserted elsewhere in the genome and hence not disrupt the *DMD* gene. For all these reasons, the reading frame rule should be applied with caution for duplications.

### Interpretation of small sequence variants

Sequencing of genomic DNA or cDNA may identify small sequence variants in the *DMD* gene. The majority of pathogenic *DMD* small variants are nonsense, frameshift or splicing variants, while missense variants are rare (Table 1).

For any small variant identified within the *DMD* gene, evidence for pathogenicity must be assessed. The interpretation of variant pathogenicity should be undertaken in accordance with international guidelines on sequence variant interpretation, currently the ACMG/AMP consensus recommendations [57]. Therefore, the variant investigation process is not described in detail here, but important considerations include: concordance with the clinical, biochemical and histological phenotype; presence/frequency in population-based cohorts (e.g. the Genome Aggregation Database [58]); previous reports in association with dystrophinopathy, including checking the *DMD* locus specific databases (Leiden Open Variation Database, LOVD [3], which includes information on whether a given variant has been associated with DMD, BMD and/or other phenotypes; UMD-DMD France database [59]) and ClinVar [60]; any RNA or protein functional studies in the literature or for the specific patient; in silico predictions including conservation across species, predicted consequence of amino acid substitutions (for missense variants) and splicing algorithms.

#### Frameshift variants

Frameshift variants typically lead to the generation of multiple premature stop codons, leading to (variably) reduced levels of full length transcripts and translational failure, therefore, being causative of a dystrophinopathy, mostly a DMD phenotype.

#### Nonsense variants

Nonsense variants are single nucleotide substitutions leading to a nonsense codon instead of an amino acid-specific codon; again, they are generally pathogenic as a result of (variably) reduced levels of full length transcripts and translational failure, mostly leading to a DMD phenotype.

However, a number of exceptions are on record of in-frame exon skipping associated with some frameshifting or nonsense variants (also rare cases of missense or even synonymous variants) weakening the splicing recognition sequences (i.e. exon splicing enhancers or ESEs), thereby changing the severity of the phenotype [50, 61]. Also, at the extreme 3′ end of the gene, nonsense variants may lead to a milder phenotype than expected for reasons not completely understood [62, 63]. At the extreme 5′ end of the gene, alternative translation initiation beginning in *DMD* exon 6 can reduce the expected ﻿severity of *DMD* nonsense variants lying within the first few exons of the gene [52, 64].

#### Splicing variants

Genomic variants located in the canonical splice sites, acceptor (positions –1 and –2) and donor (positions +1 and +2), will almost invariably lead to aberrant splicing as they affect strongly conserved dinucleotides that define exon–intron boundaries. Bioinformatics in silico tools can be used to support the prediction that the splice site is abolished (the splice site is generally no longer recognised or its score is drastically reduced). Variants may also occur in less conserved regions of the splicing consensus sequences (e.g. the final two nucleotides of exons, splice donor site positions +3 to +6, splice acceptor polypyrimidine tract and the branchpoint). In silico predictions for these variants are less precise. It is recommended to use multiple software programs (at least three) to give a consensus prediction. There is no consensus on the threshold cut-off value and this should be adapted (usually from 5 to 15%) depending on the prediction tool [65, 66]. These tools are also able to predict newly created cryptic splice sites. In all cases, these are only predictions. A splice site variant can have different effects on transcripts (single or double exon skipping, use of de novo splice sites, activation of a cryptic splice site and pseudo-exon inclusion, retention of introns or even a combination of these). Therefore, if possible, muscle RNA analysis should be performed or recommended for reading frame determination and clinical interpretation, which can be supported in this case by the immunohistochemical analysis of dystrophin.

Apart from the loss or the gain of a splice site, determining whether other exonic or intronic DNA variants can lead to aberrant splicing is more complex. In theory any intronic or exonic (nonsense, missense, synonymous) sequence variation might affect splicing by changing (creation or disruption) splicing regulatory elements. However, in view of the current absence of reliable bioinformatics programs to predict these *cis*-acting elements, muscle RNA analysis in the patient or functional assays are required for the classification of such variants. The above-mentioned exon skipping-associated nonsense variants and the reported case of a pseudo-exon activation [67] are examples of such mechanisms.

#### Missense variants

Pathogenic missense variants in the *DMD* gene are rare and can cause DMD, BMD or X-linked cardiomyopathy. Variants leading to amino acid substitutions should be checked for their predicted effect on protein structure and function by suitable algorithms, such as SIFT [68] and PolyPhen-2 [69]. With a huge, still not fully characterised, protein like dystrophin, the limited power of such predictions should be recognised. A significant proportion of reported missense variants are located in the ABD1 of dystrophin for which the crystal structure has been determined [70]; these frequently lead to a BMD phenotype. Another subset of missense variants lie in the ZZ domain of the C-terminal region of the protein; these variants are typically reported in patients with a severe Duchenne phenotype in spite of showing persistence of dystrophin staining in muscle in some cases [71]. The interpretation of previously unreported missense variants or variants located in other protein domains is more complicated and currently relies only on in silico analyses. The possibility of splicing effects, as discussed above, should also be considered.

### Reporting results

#### Introduction/general comments

Reporting genome/gene variants is the crucial final step of genetic diagnosis, and the report represents a lifelong durable document for the patient and his/her relatives, that rarely needs to be revised or updated. The report should be comprehensible on its own, written in clear (though specific) language and provide a fully interpretative and authoritative answer to the clinical question, thus containing all necessary information for the reader.

General guidelines on reporting results of diagnostic genetic testing, including guidance on report format and essential information, have previously been published to aid harmonisation of European reporting practice [72]. Therefore, these guidelines should be adhered to and further general information is not provided here.

When reporting genetic testing of the *DMD* gene, an appropriate reference sequence accession number (including version where applicable) must be included (refer to variant annotation section above). The analysed gene regions (exons, full coding sequence, etc.) and methods used (referring to commercial kit reference and version numbers if applicable) should be specified. If an NGS method is used, any exons/regions not covered should be outlined and the alternative/additional methods used (e.g. Sanger sequencing) to sequence the uncovered exons should be indicated.

HGVS variant nomenclature is the gold standard for variant annotation on reports (refer to variant annotation section above). However, for deletions or duplications of one or more exons of the *DMD* gene, a description with respect to exons (e.g. deletion of exons 2–6) is more easily understandable to the report reader, and is, therefore, recommended. HGVS nomenclature must be used for small sequence variants and may optionally be included for CNVs (along with the description with respect to exons).

### Diagnostic reports

#### Variant detected

When reporting a variant, it is recommended to describe the variant type (deletion, duplication, frameshifting, nonsense, missense, splicing, other complex variants) to impart clarity to the report. The zygosity should also be stated, i.e. hemizygous or heterozygous. It is essential to report the pathogenicity of the variant, i.e. pathogenic, likely pathogenic or uncertain significance. Variants classified as benign or likely benign are not routinely included in patient reports.

Rare and inherited disease genomic reporting practice for variants of uncertain significance is controversial and varies between centres [73]. Therefore, review of this topic for wider genetics and genomics reporting is beyond the scope of these guidelines. However, for diagnostic tests for dystrophinopathy, where no pathogenic or likely pathogenic variant is detected, variants of uncertain significance should generally be reported, as it may be possible to further investigate pathogenicity in a number of ways, including muscle immunocytochemistry, muscle RNA analysis and segregation studies.

For deletions of one or more exons, the predicted effect on the reading frame should also be stated, underlining that it is a *prediction without diagnostic value*. This may optionally be stated for duplications, although with greater caution for reasons discussed previously. If the *DMD* mRNA profile has been characterised, the effect on the mRNA should be reported including the impact on the reading frame.

For a pathogenic or likely pathogenic variant, state that the result confirms, or is consistent with, the diagnosis of dystrophinopathy. If the patient’s phenotype is consistent with the predicted severity of the variant, then the report can specifically state that the result confirms, or is consistent with, either DMD or BMD. If the phenotype does not appear consistent with the predicted severity of the variant, the diagnosis of dystrophinopathy is still confirmed, but the report may optionally comment that further analysis could be undertaken (e.g. muscle RNA analysis).

When reporting a variant of uncertain significance, the report should state that the result neither confirms nor excludes a diagnosis of dystrophinopathy. The possibility of further investigations (such as testing other family members and/or muscle biopsy for immunocytochemistry and RNA analysis) should also be included in the report.

Reports should also suggest referral for genetic counselling.

The same considerations apply to reporting results of diagnostic testing in manifesting females as for males, although in the absence of information on skewed X-inactivation or RNA analysis, it is not possible to comment on the genotype/phenotype correlation and genetic prognosis.

#### No variant detected

The report should state that the result neither confirms nor excludes a diagnosis of dystrophinopathy (or convey this meaning using alternative phrasing). The sensitivity of the test(s) must be given. Guidance on test sensitivity is included in the ‘Carrier test reports’ section below; this will be very high (~99%) following level 2 testing and higher still after level 3 testing. Depending on which stage of the diagnostic algorithm (Fig. 1) the report is being issued, the possibility of further testing should be commented on. This applies equally for males and females.

### Carrier test reports

#### Variant detected

State that the variant is heterozygous (assuming this is the case).

For a pathogenic variant, state that the individual is a carrier of dystrophinopathy/DMD/BMD (as appropriate). For females proven to be carriers, the report should also state that the individual is at risk of clinical symptoms, particularly cardiomyopathy, given the known risk for carriers to develop age-dependent cardiomyopathy [74, 75] and also skeletal muscle weakness [75]. It may also be helpful to recommend clinical follow up with cardiac function surveillance.

The feasibility of prenatal diagnosis, including PGT, should be mentioned. Carrier testing and genetic counselling may also be offered to other appropriate relatives.

#### No variant detected

When the familial pathogenic variant is not present in a carrier test, state that the individual is not a carrier of the familial variant. However, an exception to this is when testing the mother of an affected individual(s) with no previous family history of dystrophinopathy (sporadic cases); in this case, state that the mother may be a germline mosaic and include an estimate of the consequent recurrence risk for offspring; up to date published data should be used to estimate this risk, currently Helderman-van den Enden et al. [5] (also refer to Mosaicism in the ‘Atypical findings’ section below); depending on local practice, prenatal diagnosis may be offered.

When the familial pathogenic variant is not known and no variant is detected, the carrier risk will be reduced. The report should state the prior and posterior carrier risks as well as the test sensitivity. The posterior risk should be calculated using Bayesian methods; detailed worked examples are included in Bridge [76] and Young [77]. Laboratories should determine test sensitivity for the tests/methods used. The following estimates of sensitivity may be appropriate and are provided for guidance, based on the European data summarised in Table 1:

- Sensitivity of analysis for deletions/duplications of one or more exons for dystrophinopathy (phenotype unknown)—78%
- Sensitivity of analysis for deletions/duplications of one or more exons for DMD—75%
- Sensitivity of analysis for deletions/duplications of one or more exons for BMD—88%
- Sensitivity of analysis for deletions/duplications of one or more exons AND sequencing of the coding region and exon/intron boundaries for dystrophinopathy (all phenotypes)—99%

Laboratories may prefer to use their own data/figures. Overall sensitivity may vary slightly between populations and is also dependent on the analytical sensitivity of the testing method(s) used.

#### Haplotype analysis

As discussed in the genetic testing strategy section, haplotype analysis may be undertaken to assess carrier status in rare cases where the familial pathogenic variant has not been identified (including when a variant of uncertain significance has been identified). Individual haplotypes should be constructed from the observed alleles and displayed in a pedigree drawing. Carrier risks should be calculated based on the segregation of high- and low-risk haplotypes, using Bayesian methods as described in worked examples published by Bridge [76] and Young [77]. In cases where a recombination is observed, calculations become more complex and may need to consider the relative likelihood of the unidentified pathogenic variant residing on either side of the recombination. A report or genetic counselling document for multiple family members is required to summarise the results, as a single individual genetic report will not be meaningful. This document should estimate the prior and posterior carrier risks, including all available pedigree information and any data on serum CK levels in women at-risk of being carriers. As stated previously, considerable caution should be exercised when reporting results of haplotype analysis; the report document should clearly state that the interpretation assumes the diagnosis of dystrophinopathy in the family is correct (i.e. that the disorder is due to a defect in the *DMD* gene).

### Prenatal diagnosis

When the familial variant is detected in a male pregnancy, reports should state that the foetus is predicted to be affected with DMD, BMD or dystrophinopathy (as appropriate). In the case of prenatal diagnosis for possible germline mosaicism risk, i.e. for a male pregnancy in a mother who has previously had a child with dystrophinopathy but is not herself a somatic carrier of the pathogenic variant, detection of the familial variant in the male foetus demonstrates that the mother is a germline mosaic. This should be documented in the report, additionally stating that the recurrence risk in future pregnancies is increased relative to the risk prior to this result but remains less than the risk of transmission (50%) from a carrier mother.

When the familial variant is not detected in a male pregnancy, it should be stated that the foetus is predicted not to be affected with DMD, BMD or dystrophinopathy (as appropriate).

Reports should state which variant(s) was/were tested for, and that maternal cell contamination was excluded.

Where prenatal diagnosis is performed by NIPD using RHDO analysis, it is important to also include test-specific performance information such as the risk of a false-positive or false-negative result.

Rare cases of invasive prenatal diagnosis by haplotype analysis should be interpreted and reported as summarised above for carrier testing by haplotype analysis.

### Atypical findings

Although simple CNVs and small variants in exons or flanking intronic sequences account for roughly 98% of pathogenic variants associated with dystrophinopathy, many types of atypical pathogenic variants do occur.

### Non-contiguous exon deletions or duplications

This is not an infrequent finding and may be identifiable by standard quantitative tests for CNVs (such as MLPA). Deletion or duplication of non-contiguous exons should always be further analysed. Following exclusion of allele drop-out due to SNPs within primer-binding sites, whole-genome CGH and/or RNA studies are helpful to define the *DMD* locus architecture and/or the RNA profile. These studies are required to confirm the pathogenic effect that the rearrangement may have on the reading frame.

### Dual *DMD* variants

In rare males (in *cis*) or in females (in *cis* or *trans*) two pathogenic *DMD* variants can be found [78]. In these cases, family segregation analysis is mandatory: in males to define their occurrence (inherited or de novo) and pathogenicity; in females to define the parental origin and the resulting genotype. RNA studies may also be helpful to define their pathogenicity.

### Variants in *DMD* alternative first exons or UTRs

CNV detection or sequencing procedures routinely analyse the muscle first exon only (first exon used for dystrophin muscle isoform or Dp427m). However, six other alternatively used first exons are represented in the dystrophin isoforms Dp427c, Dp427p, Dp260, Dp140, Dp116 and Dp71. Although not routinely analysed, other than CNV analysis of Dp427c by MLPA, pathogenic variants in the six alternative first exons have never been reported. In addition, given the prominent muscle (skeletal and cardiac) phenotype in dystrophinopathies, it is important to note that the only isoform other than Dp427m that is well expressed in striated muscle is the Dp427c [79, 80].

The entire untranslated regions (UTRs) at the 5′ or 3′ ends of the *DMD* gene are not completely explored in routine diagnostic testing. Pathogenic variants in these UTRs may occur, although very rarely. The first 200 bp of the 3′ UTR are routinely analysed by CNV analysis and sequenced in some laboratories, and indeed pathogenic variants have been reported [3].

### Contiguous gene syndromes

Contiguous gene syndromes that include the *DMD* gene are extremely rare, for example complex glycerol kinase deficiency [81]. When atypical or ‘plus’ phenotypes occur, karyotype and high-density whole-genome CGH are recommended to identify complex rearrangements and related gene/genomic breakpoints possibly involving extra-*DMD* regions and further genes. Accurate identification of these cases is extremely important to address appropriate care and therapy, for genetic counselling in the family, and for trial enrolment.

### Mosaicism

Pathogenic *DMD* variants can be present in the mosaic state, both somatic and germline.

Germline mosaicism in apparently non-carrier mothers of sporadic cases of dystrophinopathy is well described, and the recurrence risk for the at-risk haplotype has been estimated to be ~9% (although this can be refined based on variant type) [5]. Appropriate reporting of this possibility has been discussed above.

Somatic mosaicism has only been described in a few rare cases, mostly in females. Identifying these individuals is, however, extremely important for carrier identification and prenatal testing. There are some reported cases, where somatic mosaic female carriers escaped detection on analysis of blood DNA, and therefore missed accurate carrier diagnosis and prevention [82].

Males with mosaicism for *DMD* variants have also been reported, though very rarely [83, 84]. They generally present with milder symptoms of the disease, and in some cases there is genetic normalisation in muscle over time. It can be difficult to identify these male patients by routine diagnosis, because the mild clinical symptoms often differ from classical dystrophinopathy phenotypes. The high diagnostic sensitivity of NGS methods (when a high sequence-read depth approach is adopted) facilitates the detection of *DMD* mosaicism, providing more insights about these rare phenotypes and the levels of mosaicism in different tissues [85].

### Personalised medicine, therapies and clinical trials

In the last 10 years, dystrophinopathies’ stakeholders experienced a flowering of novel drugs and clinical trials [86]. These new therapeutic options greatly accelerated research into the pathophysiology of dystrophinopathies and also positively impacted on the standard of care and genetic diagnosis. Indeed, both accurate clinical outcome measures and accurate genetic profile are compulsory for clinical trial design. To date, this intense activity has only involved Duchenne muscular dystrophy, with the main therapeutic options being as follows: (i) reframing the *DMD* transcript via exon skipping which is induced by a synthetic antisense oligoribonucleotide (eteplirsen, golodirsen, casimersen and drisapersen) omitting exon 51, 53, 45 or 44 respectively; (ii) ribosomal read-through of nonsense variants (ataluren), which is based on the known low specificity of tRNAs in cognate codon recognition; (iii) gene therapy based on minigenes which encode shortened forms of dystrophin. These new trials and treatments strongly impact on genetic testing, since the genetic result is needed to make patients eligible and enrollable in these trials.

Information on drug eligibility is beyond the obligations of a diagnostic genetic report for dystrophinopathy and currently it is preferable not to include this in the report. However, mention of relevant information is acceptable where this is in accordance with local policy and current practice.

### Incidental findings

Increasingly, genome-wide testing is being undertaken for many different clinical indications, and this may identify variants in the *DMD* gene in patients where a clinical diagnosis of dystrophinopathy has not been considered or suspected. This includes whole-genome sequencing, whole-exome sequencing, gene panel analysis and array CGH. In particular, the wide use of array CGH in many centres is identifying cases of deletions or duplications of one or more exons of the *DMD* gene (e.g. ~1 in 500 array CGH tests in one centre, CF personal communication), often in children with developmental delay or learning difficulties, but also for a range of other scenarios including prenatal array CGH testing.

These ‘incidental’ findings of hemizygous (males) or heterozygous (females) *DMD* variants must be interpreted with caution.

CNVs of potential clinical significance identified by array CGH are reported as part of routine practice. Published guidelines on reporting of incidental findings from NGS panels, exome or genome sequencing should be referred to when considering reporting *DMD* variants identified by these methods [87].

### Male patients

In a male patient in whom there has been no clinical suspicion of dystrophinopathy, the prior probability of a *DMD* variant being pathogenic is lower than in a patient referred with classical symptoms of dystrophinopathy and highly elevated serum CK levels. Similarly, the prior probability that a pathogenic *DMD* variant is associated with high penetrance and expressivity is also lower.

Although deletions of one or more exons of the *DMD* gene are almost always pathogenic, several cases with minimal phenotypic expressivity have been reported; examples include asymptomatic males aged over 60 years with deletions of exons 48–51, 48–53 and 45–51 [88, 89] or a single exon 48 deletion [90]. Therefore, when reporting deletions identified as ‘incidental’ findings, it is recommended to state that their penetrance and expressivity is uncertain.

For duplications of one or more exons, in addition to the uncertainty regarding penetrance and expressivity, the location and orientation of the duplication is normally unknown (e.g. if detected by array CGH). Therefore, although any duplication is most likely to be a direct tandem duplication within the *DMD* gene, without additional analysis this is not certain; the duplicated region could, for example, be on an autosome and hence not disrupt the *DMD* gene (e.g. case of *DMD* exons 45–51 duplication located on chromosome 17; CF personal communication). Therefore, when a duplication is detected as an ‘incidental’ finding, this cannot demonstrate a diagnosis of dystrophinopathy and further investigations are indicated (see below).

Small *DMD* gene variants should be interpreted with similar caution to exonic deletions. Therefore, it is recommended to state that their penetrance and expressivity is uncertain.

In all cases, further investigations are recommended. The simplest follow-up to further investigate a possible diagnosis of dystrophinopathy is normally to recommend clinical and biochemical (serum CK measurement) assessment. Some centres/laboratories may be able to obtain this information prior to issuing a report, and this can be extremely helpful. If the clinical and biochemical investigations are strongly suggestive of a dystrophinopathy, then the results can then be reported in the same way as for routine diagnostic referrals. Conversely, if clinical and biochemical assessment does not support a dystrophinopathy, then it may be possible to dismiss the variant as not associated with disease; however, caution should be exercised due to the possibility of variable penetrance and expressivity within a family [91]. When the clinical significance of a variant remains uncertain, family studies may be helpful, typically beginning with testing the patient’s mother (and father for duplications where the location of the duplication is unknown) to determine whether the variant has arisen de novo. Testing other at-risk male relatives, following clinical and biochemical assessment, can also be informative if they are found to have the variant. Of course, any family studies should be supported by appropriate genetic counselling. Finally, for duplications, further molecular testing to determine the location and orientation may be possible, for example by long-range PCR, FISH or RNA analysis.

In cases where the *DMD* variant is pathogenic and clinical assessment supports a diagnosis of dystrophinopathy, this may explain the patient’s symptoms if they are known to be associated with dystrophinopathy. For example, for a male patient referred with developmental delay, this may be a gross motor delay entirely consistent with a classic dystrophinopathy phenotype. Also, for a male patient with learning difficulties, the *DMD* gene variant may be responsible as intellectual disability or cognitive impairment has been reported in a proportion of males with dystrophinopathy, particularly when the pathogenic variant is towards the 3′ end of the gene (exon 45 or beyond; likely due to impact on short dystrophin isoforms expressed in the brain) [92, 93].

### Female patients

For females in whom there has been no clinical suspicion of dystrophinopathy, again the prior probability of a *DMD* variant being pathogenic is much lower than in a patient referred with symptoms suggestive of dystrophinopathy. However, the situation is further complicated by the fact that DMD/BMD carriers are likely to be asymptomatic and so there are unlikely to be any clinical symptoms to correlate with the *DMD* genotype.

In general, the same cautionary notes regarding pathogenicity, penetrance and expressivity of deletions, duplication and small variants in the *DMD* gene apply as summarised for ‘incidental’ findings in males above. Therefore, even for variants that are normally expected to be pathogenic (such as deletions of one or more exons), it is recommended to state that the penetrance and expressivity is unknown.

Further investigations are recommended for females in whom a heterozygous *DMD* variant is identified as an ‘incidental’ finding. Clinical and biochemical (serum CK measurement) assessment may be helpful, but, unless this is suggestive of a manifesting female, these investigations are unlikely to determine whether the variant will be associated with a dystrophinopathy phenotype in males. Therefore, parental testing is recommended whenever possible to determine the inheritance pattern. In rare cases, the variant will have been inherited from the proband’s asymptomatic father; for example, Nguyen et al. [94] reported two such families, one with deletion of *DMD* exon 48 and the other with deletion of exons 3–9, indicating that these deletions have low penetrance and expressivity in these two families. Referral to a Clinical Geneticist and/or Genetic Counsellor is also recommended. If the variant has been inherited from one of the parents, the geneticist/counsellor will be able to take a detailed family history to review whether this is suggestive of a dystrophinopathy in the family. Testing other at-risk male relatives may then be informative, as discussed above for male patients. Again, for duplications, further molecular testing to determine the location and orientation may be possible.

### Prenatal samples

*DMD* deletions or duplications may also be detected as incidental findings in foetal samples during pregnancy by prenatal array CGH testing. As described above, deletions may be associated with reduced penetrance and/or expressivity, and there may be further uncertainty for duplications. Therefore, segregation/family studies are always needed to define pathogenicity in the family. In some cases, the clinical significance and/or pathogenicity of the *DMD* variant may remain uncertain.

### Historical results

In known dystrophinopathy families, previous genetic testing at the *DMD* locus may date as far back as the mid-1980s. Results of these historical tests are often difficult to interpret and/or understand, are unlikely to be as definitive as current testing, and in many cases were carried out in research laboratories or other non-accredited laboratories. Therefore, when a new referral arises in such a family and when no recent genetic testing has been undertaken, it is recommended that genetic testing be repeated using current techniques.

## Discussion

These guidelines constitute an update of previous European guidelines [7] and describe best practice for diagnostic and family genetic testing and reporting for dystrophinopathies. Updated guidelines were required, due to the application of new genetic technologies in diagnostics and the availability of new personalised drugs since 2010.

Draft updated recommendations prepared by the authors were disseminated to a global network of molecular geneticists and clinicians (from 135 laboratories who have participated in the EMQN external quality assessment scheme for DMD and UK diagnostic laboratories) for consultation and amendments. Approximately 20 comments were received and appraised by the authors; all were minor and the guidelines were modified to incorporate the majority of the suggestions.

Key updates and additions since the 2010 guidelines include: use of NGS in diagnostics; non-invasive prenatal diagnosis; guidance on sequence variant annotation and interpretation; atypical findings; implications for personalised medicine and clinical trials; and identification of *DMD* gene variants in patients where a clinical diagnosis of dystrophinopathy has not been suspected (incidental findings).
